# Indocyanine green–mediated antimicrobial photodynamic therapy as an adjunct to periodontal therapy: a systematic review and meta-analysis

**DOI:** 10.1007/s00784-021-03871-2

**Published:** 2021-03-12

**Authors:** Nasir Zeeshan Bashir, Har-Amrit Singh, Satnam Singh Virdee

**Affiliations:** School of Dentistry, University of Birmingham, Edgbaston, Birmingham, B5 7SA UK

**Keywords:** Antimicrobial photodynamic therapy, Indocyanine green, Lasers, Periodontitis

## Abstract

**Objectives:**

The aim of this systematic review and meta-analysis was to evaluate the efficacy of indocyanine green–mediated photodynamic therapy (ICG-PDT) as an adjunct to non-surgical periodontal therapy (NSPT), in the management of chronic periodontitis.

**Materials and methods:**

Four electronic databases (PubMed, Cochrane Central Register of Controlled Trials, Embase via OVID, Web of Science) were searched for randomised controlled trials comparing NSPT with ICG-PDT to NSPT without laser therapy. Primary outcome measures were changes in probing pocket depth (PPD) and clinical attachment level (CAL). Clinical outcomes were extracted and pooled from 7 eligible trials and meta-analyses conducted using mean difference with standard deviations.

**Results:**

For PPD, adjunctive ICG-PDT resulted in a mean additional reduction of 1.17 mm (95% CI: 0.67–1.66 mm) at 3 months and a mean additional reduction of 1.06 mm (95% CI: 0.54–1.57 mm) at 6 months. For CAL, adjunctive ICG-PDT resulted in a mean additional gain of 0.70 mm (95% CI: 0.17–1.23 mm) at 3 months and a mean additional gain of 1.03 mm (95% CI: 0.83–1.24 mm) at 6 months. No adverse events were reported in any studies.

**Conclusions:**

The adjunctive use of ICG-PDT in NSPT results in improved treatment outcomes at 3 and 6 months post-therapy. Further investigation is needed to evaluate variables such as different photosensitiser concentrations and adjusting parameters associated with the light source.

**Clinical relevance:**

Indocyanine green–based photosensitisers may be a novel, clinically efficacious agent for use in the management of periodontitis.

**Supplementary Information:**

The online version contains supplementary material available at 10.1007/s00784-021-03871-2.

## Introduction

Periodontitis is a chronic inflammatory condition of the periodontium that results from pathological interactions between virulent bacteria and the host response [1]. This disease, which ultimately leads to loss of periodontal attachment, is the sixth most prevalent worldwide and has been implicated in the pathophysiology of numerous other systemic inflammatory conditions such as rheumatoid arthritis, diabetes, and chronic kidney disease [1–3]. Conventional management principally involves eliminating the causative pathogens and, in doing so, arresting the inflammatory response [4]. Mechanical debridement of the root surface, with an intent to disrupt the biofilm, in combination with a meticulous oral hygiene regimen forms the cornerstone of effective periodontal therapy [5]. This approach, which is referred to as non-surgical periodontal therapy (NSPT), has been clinically proven to be effective at producing improvements in probing pocket depth (PPD) and clinical attachment level (CAL) for the majority of patients [6]. However, when disease persists, the use of adjunctive agents could be considered to enhance outcomes of non-surgical approaches [7]. A variety of adjunctive agents have been studied for their application in the management of periodontitis. Of these, antibiotics, administered systemically or locally, have demonstrated clinical effectiveness, producing anywhere from 0.40 mm reduction in PPD to in excess of 0.80 mm [7–9]. However, antibiotics are greatly limited in their applications due to the associated risks, namely, antimicrobial resistance, risk of anaphylaxis, and the requirement of high dosages when delivered systemically [10]. Alternatively, antimicrobial photodynamic therapy (aPDT) is another adjunctive periodontal treatment modality that employs the use of low-level lasers, with a photosensitiser, to generate cytotoxic free radical species [11]. These, in turn, eliminate the causative bacteria by damaging the cytoplasmic membrane, as well as bacterial DNA [11]. Given that mechanical debridement alone cannot eliminate all pathogens, due to factors such as complicated root anatomy, the presence of furcation defects, and inaccessible reservoirs of bacteria in the cementum and dentine tubules, it would appear that aPDT may convey benefits in the management of periodontal disease [12–14].

Typically, aPDT has been assessed in the context of conventional photosensitising agents, such as toluidine blue and methylene blue. These conventional agents act through photochemical means and appear to be of limited clinical benefit [15–18]. However, contemporary photosensitising agents have now been developed, with the promise of greater efficacy in the management of periodontal disease. One such photosensitising agent that is commonly investigated is indocyanine green (ICG). This anionic photosensitiser has a peak absorption higher than conventional agents and displays its effects primarily through photothermal activity, which is in contrast to the conventional agents that exert their effects through photochemical means [19–21]. Moreover, ICG has in vitro shown to be taken up significantly in periodontal pathogens, namely, *Porphyromonas gingivalis* and *Aggregatibacter actinomycetemcomitans*, and is therefore highly efficacious in eliminating microorganisms highly associated with periodontitis [22, 23]. Furthermore, ICG-mediated photodynamic therapy (ICG-PDT) is effective in eliminating antimicrobial-resistant strains of commonly occurring bacterial species [23]. Collectively, this evidence indicates that ICG-PDT may provide clinical benefits in scenarios which are outside the remit of conventional antimicrobials [24]. However, there is no overall consensus on the clinical benefits of ICG-PDT, and therefore, robust synthesis of the literature evaluating ICG-PDT is required in order to establish whether it may be beneficial for the management of periodontal disease, and current evidence is based solely upon outdated conventional photosensitising agents [25, 26]. To the authors’ knowledge, there are at present no existing systematic reviews evaluating the efficacy of ICG-PDT as an adjunct to NSPT in improving treatment outcomes.

The aim of this systematic review and meta-analysis was to compare the clinical efficacy, with respect to PPD and CAL, of ICG-PDT-supported NSPT to that of conventional non-adjunctive NSPT in patients diagnosed with periodontitis.

## Materials and methods

### Protocol and registration

The protocol for this study was prospectively registered in the International Prospective Register of Systematic Reviews, PROSPERO (CRD42020197738). This review is reported according to PRISMA guidelines.

### Eligibility criteria

Randomised controlled trials directly comparing the clinical effectiveness of ICG-PDT-supported NSPT to that of non-adjunctive NSPT were included in this review. Studies were required to have a minimum follow-up period of 3 months, evaluated outcomes in systemically healthy non-smoking adult patients (≥18 years of age) with periodontitis, which was defined as PPD ≥ 5 and/or CAL ≥ 4 [27], who received no concurrent antimicrobial therapy.

Studies were excluded if they evaluated outcomes in implants and surgical periodontal therapy or if they were conducted in animals. No restrictions were placed on the studies according to the date of publication, but only those in English were selected to avoid errors in interpretation.

### Information sources and search

A search strategy was developed by expanding on the key terms of ‘indocyanine green’ and ‘periodontitis’ using synonyms, key phrases, indexed databases, and authors’ knowledge. The search terms were combined using Boolean operators (‘AND’, ‘OR’) to account for sensitivity and specificity. On 7 August 2020, four electronic databases were searched from inception to 30 June 2020: PubMed, Cochrane Central Register of Controlled Trials, Embase via OVID, and Web of Science. Additionally, reference list follow-ups of all included studies were conducted. The search strategy for PubMed is outlined in Table 1.

**Table 1:** Summary of PubMed search strategy

Input query	Articles returned
((("indocyanine green"[MeSH Terms] OR ("indocyanine"[All Fields] AND "green"[All Fields])) OR "indocyanine green"[All Fields]) OR "emundo"[All Fields]) AND ("periodont*"[All Fields] OR (((("gingival diseases"[MeSH Terms] OR ("gingival"[All Fields] AND "diseases"[All Fields])) OR "gingival diseases"[All Fields]) OR ("gum"[All Fields] AND "disease"[All Fields])) OR "gum disease"[All Fields]))	52

### Study selection

The title and abstracts of studies were initially screened independently by two reviewers (NZB and HS) in accordance with the aforementioned eligibility criteria. Thereafter, articles underwent full-text analysis in a similar manner with reasons for exclusion documented. Discrepancies between the reviewers were settled through independent adjudication by a third review author (SSV).

### Data extraction and items

Data from the included studies on the author, year, study setting, age range of participants, sample size, treatment protocols, and review periods were extracted into a custom-designed spreadsheet made in Microsoft Excel (2019). A standardised data sheet was pre-piloted and then implemented for data extraction by a single reviewer (NZB). The second reviewer (HS) verified the accuracy of data obtained from the studies.

### Risk of bias

The risk of bias of the included studies was evaluated using the criteria outlined in the *Cochrane Handbook for Systematic Reviews of Interventions* [28]. The following parameters were assessed: random sequence generation, allocation concealment, blinding of participants and personnel, blinding of outcome assessment, incomplete outcome data, selective reporting, and other bias. Studies evaluated to be at high risk for any parameter were deemed to be at high risk of bias overall. All studies were initially incorporated for quantitative synthesis, and then sensitivity analyses were conducted to assess the contribution of each study to the totality of the evidence. This allowed for assessment of the impact of high-risk trials on the overall effect size.

### Summary of measures

Qualitative synthesis was conducted for all studies that met the inclusion criteria, and key characteristics of each study were summarised and presented in both text and table format.

Quantitative data were then extracted to allow for meta-analyses. The primary outcome being assessed was change in PPD and CAL, and risk of adverse events with ICG-PDT was assessed as a secondary outcome measure.

### Data synthesis

Inter-reviewer agreement for screening and inclusion of articles was assessed via Cohen’s kappa scores.

Meta-analyses were conducted for treatment outcomes at 3 months and 6 months. Data from the included studies were pooled, using mean difference (mm) with standard deviations (SDs). If data were presented in an unclear format or ambiguous in presentation, the authors were contacted for further clarification. If SDs were missing, these were imputed from the following formula for variance (Var): Var _change from baseline_ = Var _baseline_ – Var _end_ – (2 * *r* * SD _baseline_ * SD _end_) (correlation [*r*] of 0.5 was assumed) [7, 29]. The secondary outcome measure, adverse events, was assessed through calculation of odds ratios.

Statistical heterogeneity was assessed through Cochran’s Q chi-squared testing and calculation of the *I*^2^ index. In accordance with the *Cochrane Handbook for Systematic Reviews of Interventions*, *I*^2^ values between 0 and 40% were deemed as not representing significant heterogeneity, and values above 40% were considered to represent significant heterogeneity. Data were pooled using both a fixed-effect model and a random effects model, and if significant heterogeneity was identified, the findings from the random effects model were presented. Forest plots were generated to illustrate the findings of the meta-analyses.

Meta-regressions would be conducted if there were an adequate number of studies (10 or more).

Risk of bias across studies (publication bias) would be evaluated through generation of funnel plots and subsequent Egger’s tests, if there were an adequate number of studies (10 or more).

Sensitivity analyses were conducted to assess the contribution of each individual study to the totality of the evidence.

All analyses were programmed in Stata version 16.0.

## Results

### Selected studies

**Fig. 1:**
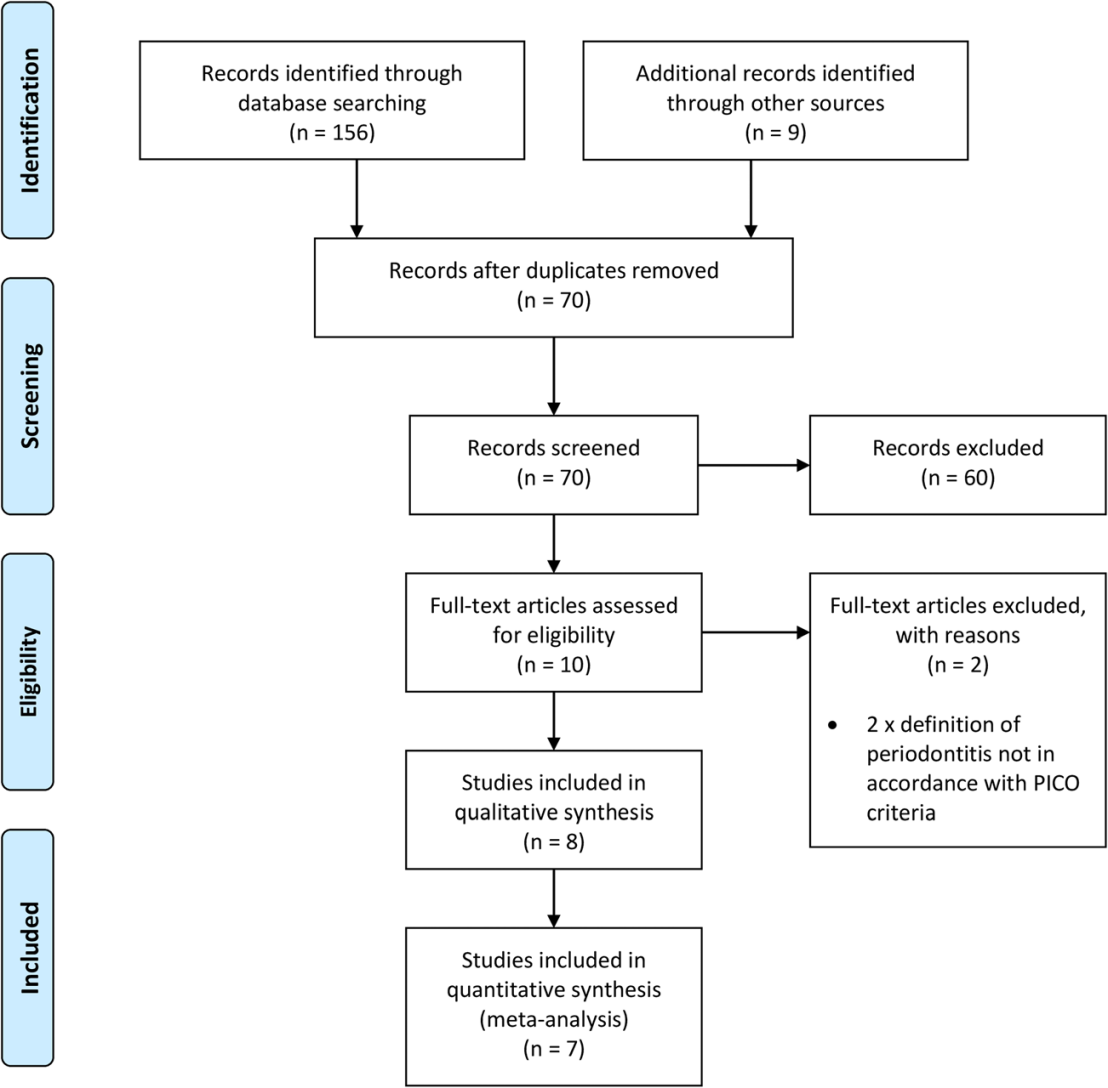
PRISMA flowchart outlining study selection process

The study selection process is outlined as a PRISMA flowchart in Fig. 1. The initial search returned 165 articles, of which, 95 articles were identified as duplicates. The remaining 70 articles were screened according to the title and abstract, and 60 were excluded. The remaining 10 studies underwent full-text analysis, of which, 8 met the inclusion criteria. Of the 8 included studies, 7 were suitable for meta-analyses. Inter-reviewer agreement for the study selection process was assessed as ‘excellent’, indicated by Cohen’s kappa scores of 1.00 [30]. The studies excluded at full-text analysis, with reasons for exclusion are presented in Table 2.

**Table 2:** Studies excluded at full-text analysis

Study	Reason for exclusion
Hill et al. 2019 [31]	Definition of periodontitis not in accordance with inclusion criteria
Niazi et al. 2020 [32]	Definition of periodontitis not in accordance with inclusion criteria

### Study characteristics

#### Study design and demographics

The included trials, and the characteristics of these studies, are presented in Table 3. Briefly, all included studies were randomised controlled trials, with five split-mouth designs [33–35, 39, 40] and three parallel-arm design [36–38]. All trials used both ultrasonic and hand instruments for NSPT, except for one, which simply stated the use of a ‘scaling tip’ [33]. All trials implemented a control group which involved no laser therapy. Two of the trials also implemented an additional group, which involved laser therapy but without the use of a photosensitising agent. Meta-analyses were only conducted comparing NSPT with ICG-PDT to NSPT with no adjunctive therapy. Meta-analyses were not conducted to compare NSPT with ICG-PDT to NSPT with laser therapy without photosensitising agents, as this was not outlined in the initial review protocol.

**Table 3:** Characteristics of included studies

Study	Disease definition	Age range (years)	Test group (*n*)	Test group protocol	Control group (*n*)	Control group protocol	Outcomes evaluated at
Chiang et al. 2020 [33]	≥ 5mm PPD, ≥ 5mm CAL, bleeding on probing	20–82	(22)	Following NSPT (‘scaling tip’), 0.1% ICG was administered. Pocket was irrigated with saline to remove excess. Diode laser (810nm/0.5 + 0.2W) was used to activate the dye from various distances for 30s (anterior teeth + premolars) or 50s (molars). A second round was repeated 4–7 days later. A third round was repeated if bleeding or soreness persisted	(22)	NSPT only	Baseline 4–6 weeks 3 months
Gandhi et al. 2019 [34]	≥ 5mm PPD	30–60	(30)	Following NSPT (ultrasonic scaling + hand instruments), ICG was administered. Pocket was irrigated with saline to remove excess. Diode laser (810nm/0.1W) was used to activate the dye for 60s	(30)	1) NSPT only 2) NSPT with laser therapy, but no photosensitising agent	Baseline 1 month 3 months 6 months 9 months
Joshi et al. 2020 [35]	≥ 5mm PPD, ≥ 3mm CAL	30–60	(29)	Following NSPT (ultrasonic scaling + hand instruments), 1mg/ml ICG was administered. Pocket was irrigated with distilled water to remove excess. Diode laser (810nm/0.2W) was used to activate the dye for 30s	(29)	NSPT only	Baseline 3 months
Monzavi et al. 2016 [36]	≥ 5mm PPD, bleeding on probing	35–55	(25*)*	Following NSPT (ultrasonic scaling + hand instruments), 1mg/ml ICG was administered. Diode laser (810nm/0.2W) was used to activate the dye from various distances for 40s. Repeated after 7, 17, and 27 days	(25)	NSPT with physiological serum and an off laser	Baseline 1 month 3 months
Raut et al. 2018 [37]	≥ 5mm PPD, ≥ 4mm CAL	30–55	(25)	Following NSPT (ultrasonic scaling + hand instruments), 5mg/ml ICG was administered. Pocket was irrigated with distilled water to remove excess. Diode laser (810nm/0.8W) was used to activate the dye for 60s	(25)	NSPT with physiological serum and an off laser	Baseline 6 months
Sethi et al. 2019 [38]	≥ 5mm PPD, ≥ 4mm CAL	30–55	(15)	Following NSPT (ultrasonic scaling + hand instruments), 5mg/ml ICG was administered. Pocket was irrigated with saline to remove excess. Diode laser (810nm/0.8W) was used to activate the dye for 60s	(15)	NSPT only	Baseline 3 months
Shingnapurkar et al. 2016 [39]	≥ 5mm PPD	25–55	(30)	1 week following NSPT (ultrasonic scaling + hand instruments), 1mg/ml ICG was administered. Patient rinsed with water to remove excess. Diode laser (810nm/0.2W) was used to activate the dye for 30s	(30)	NSPT with dye and a sham laser	Baseline 1 month 3 months
Srikanth et al. 2015 [40]	≥ 5mm PPD	30 – 55	(*30*)	Following NSPT (ultrasonic scaling + hand instruments), 5mg/ml ICG was administered. Diode laser (810nm/0.7W) was used to activate the dye for 5s	(30)	1) NSPT only 2) NSPT with laser therapy, but no photosensitising agent	Baseline 1 week 4 weeks 12 weeks 24 weeks

#### Disease definition

All studies used diagnostic terminology outlined in the 1999 Periodontal Disease Classification System [41]. Seven of the studies evaluated patients with ‘chronic periodontitis’, and 1 study evaluated patients with ‘refractory periodontitis’.

#### Outcome assessment

The data for mean changes in PPD and CAL for all included studies are presented in Table 4, with the key findings summarised. All studies reported on changes in PPD and CAL, and these were extracted to allow for meta-analyses. Not all studies reported outcomes at both 3 months and 6 months, with 6 studies reporting outcomes at 3 months and 2 studies reporting outcomes at 6 months. One study presented all data in a graphical format, and the authors were contacted for numerical data that would allow for meta-analysis [33]. These data were not provided, and therefore, this study was not eligible for quantitative synthesis. One study evaluated outcomes at 1 week, 4 weeks, 12 weeks, and 24 weeks post-therapy; the outcomes at 12 weeks and 24 weeks were pooled into the meta-analyses for outcomes at 3 months and 6 months, respectively [40]. All studies explicitly stated that outcomes were assessed at the site-specific level, except for one trial which did not make it clear if full-mouth or site-specific outcomes were being reported on [36].

**Table 4:** Observed changes in outcomes in included studies

Study	Outcomes measured at	Mean reduction in PPD ± SD (mm)	Mean gain in CAL ± SD (mm)	Key findings
Gandhi et al. 2019 [34]	3 months	NSPT + ICG-PDT = 2.79 ± 0.63 NSPT = 1.19 ± 0.82	NSPT + ICG-PDT = 2.69 ± 0.68 NSPT = 1.16 ± 1.09	NSPT with ICG-PDT was significantly more efficacious than NSPT alone
	6 months	NSPT + ICG-PDT = 1.74 ± 1.11 NSPT = 0.75 ± 0.90	NSPT + ICG-PDT = 1.63 ± 1.43 NSPT = 0.74 ± 1.19	NSPT with ICG-PDT was significantly more efficacious than NSPT alone
Joshi et al. 2020 [35]	3 months	NSPT + ICG-PDT = 2.36 ± 0.37 NSPT = 2.10 ± 0.35	NSPT + ICG-PDT = 2.34 ± 0.37 NSPT =2.10 ± 0.35	NSPT with ICG-PDT was significantly more efficacious than NSPT alone
Monzavi et al. 2016 [36]	3 months	NSPT + ICG-PDT = 2.54 ± 0.29 NSPT = 0.63 ± 0.79	NSPT + ICG-PDT = 1.36 ± 0.77 NSPT = 1.55 ± 0.76	NSPT with ICG-PDT was significantly more efficacious than NSPT alone
Raut et al. 2018 [37]	6 months	NSPT + ICG-PDT = 2.51 ± 0.40 NSPT = 1.00 ± 0.62	NSPT + ICG-PDT = 1.68 ± 0.82 NSPT = 0.72 ± 0.75	NSPT with ICG-PDT was significantly more efficacious than NSPT alone
Sethi et al. 2019 [38]	3 months	NSPT + ICG-PDT = 1.86 ± 1.11 NSPT = 0.70 ± 0.60	NSPT + ICG-PDT = 1.41 ± 1.20 NSPT = 0.99 ± 0.77	NSPT with ICG-PDT was significantly more efficacious than NSPT alone
Shingnapurkar et al. 2016 [39]	3 months	NSPT + ICG-PDT = 2.90 ± 0.75 NSPT = 1.60 ± 0.78	NSPT + ICG-PDT = 2.53 ± 0.75 NSPT = 1.23 ± 1.22	NSPT with ICG-PDT was significantly more efficacious than NSPT alone
Srikanth et al. 2015 [40]	3 months	NSPT + ICG-PDT = 2.91 ± 0.73 NSPT = 2.07 ± 0.27	NSPT + ICG-PDT = 2.44 ± 0.80 NSPT = 1.50 ± 0.49	NSPT with ICG-PDT was significantly more efficacious than NSPT alone
	6 months	NSPT + ICG-PDT = 2.74 ± 0.52 NSPT = 2.06 ± 0.17	NSPT + ICG-PDT = 2.47 ± 0.40 NSPT = 1.40 ± 0.49	NSPT with ICG-PDT was significantly more efficacious than NSPT alone

### Risk of bias

**Fig. 2:**
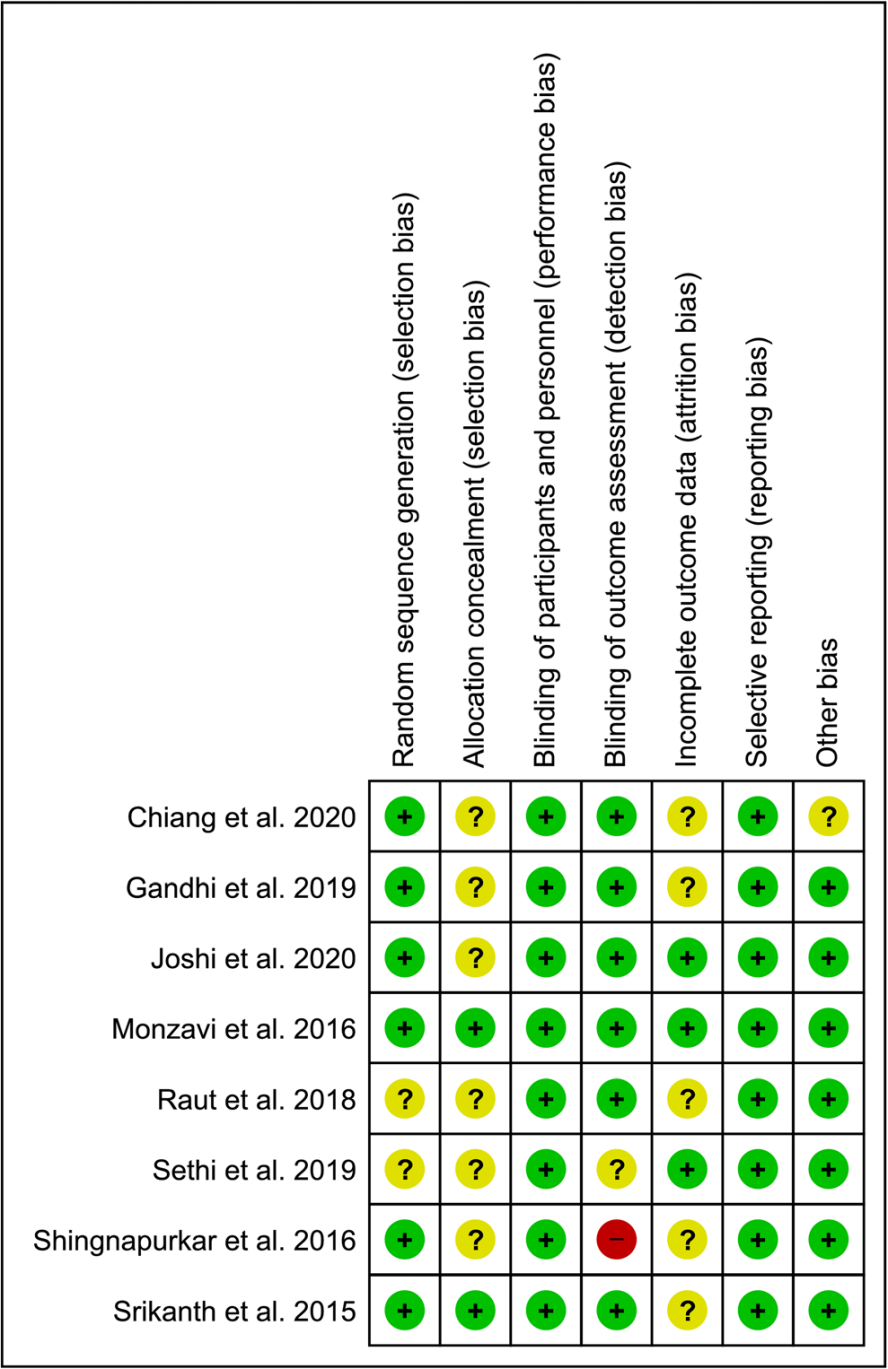
Risk of bias summary

A risk of bias summary for all included studies is provided in Fig. 2. Of the eight trials, one was deemed to be at low risk of bias, one at high risk of bias, and the remaining six at unclear risk of bias. Of the six trials at unclear risk of bias, one was not suitable for quantitative synthesis [33].

Across the seven trials suitable for quantitative synthesis, the parameters for blinding of participants and personnel, selective reporting, and other bias were assessed to be at low risk of bias for all trials. The next most common findings were five trials which were assessed as being at unclear risk of bias for allocation concealment [34, 35, 37–39], four trials assessed as being at unclear risk of bias for incomplete outcome data [34, 37, 39, 40], two trials assessed as being at unclear risk of bias for random sequence generation [37, 38], and one trial assessed as being at unclear risk of bias for outcome assessment [38]. For all of these unclear risks of bias findings, there was insufficient detail in the reporting of these parameters within the articles themselves, which meant it could not be determined whether the protocols used were of adequate quality in reducing the risk of bias, leading to unclear assessments for these trials.

Furthermore, one trial was assessed as being at high risk of bias for blinding of outcome assessment [39], as the authors explicitly stated that the personnel assessing outcomes were not blinded to the treatment received.

### Synthesis of results

#### Probing pocket depth

**Fig. 3:**
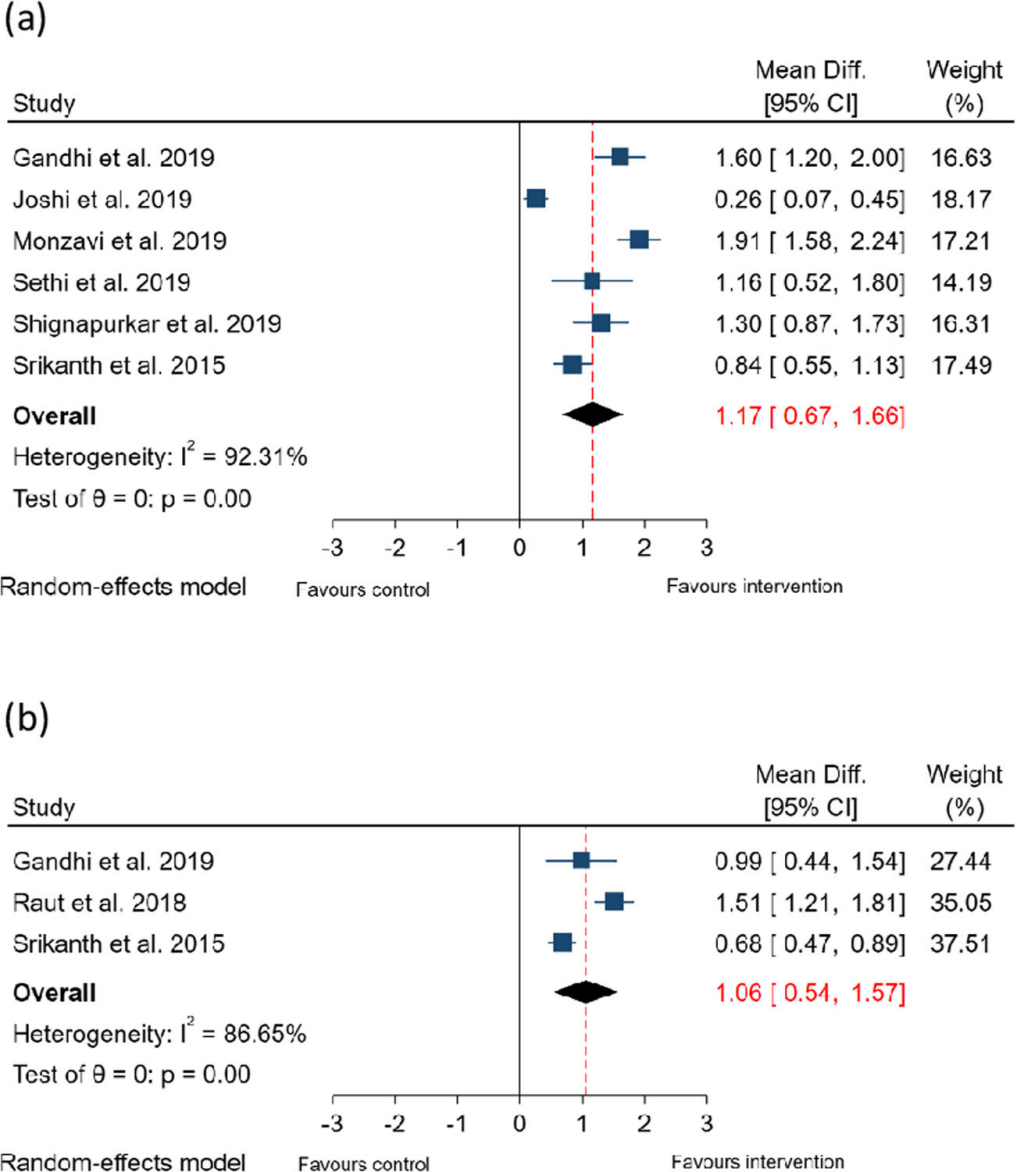
Forest plots summarising effect of ICG-PDT on PPD. (**a**) Effect of ICG-PDT on PPD reduction at 3 months and (**b**) Effect of ICG-PDT on PPD reduction at 6 months

Presented in Fig. 3 are forest plots summarising the findings of the meta-analyses for reduction in PPD.

Compared to NSPT without laser therapy, the adjunctive use of ICG-PDT resulted in a mean additional reduction in PPD of 1.17 mm (95% CI: 0.67–1.66 mm, *p* < 0.001) at 3 months and of 1.06 mm (95% CI: 0.54–1.57 mm, *p* < 0.001) at 6 months. Studies evaluating outcomes at 3 months and 6 months demonstrated significant heterogeneity (*I*^2^ = 92% and 87%, respectively), so the findings from the random effects model are presented.

#### Clinical attachment level

**Fig. 4:**
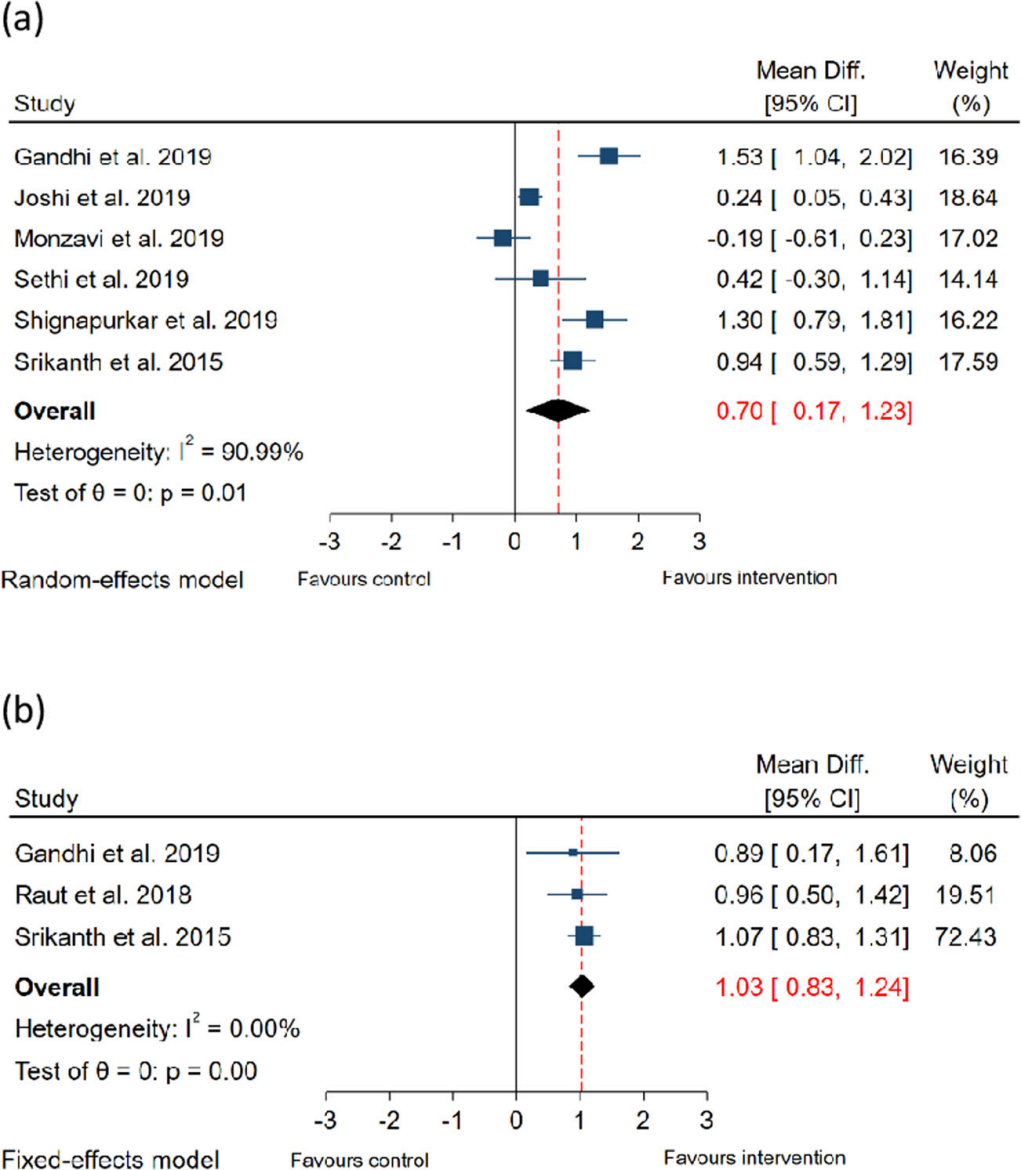
Forest plots summarising effect of ICG-PDT on CAL. (**a**) Effect of ICG-PDT on CAL gain at 3 months and (**b**) Effect of ICG-PDT on CAL gain at 6 months

Presented in Fig. 4 are forest plots summarising the findings of the meta-analyses for gain in CAL.

Sub-group meta-analyses were conducted for outcomes at 3 months and 6 months post-therapy. Compared to NSPT without laser therapy, the adjunctive use of ICG-PDT resulted in a mean additional gain in CAL of 0.70 mm (95% CI: 0.17–1.23 mm, *p* < 0.001) at 3 months and of 1.03 mm (95% CI: 0.83–1.24 mm, *p* < 0.001) at 6 months. Studies evaluating outcomes at 3 months demonstrated significant heterogeneity (*I*^2^ = 90%), so the findings from the random effects model are presented. Studies evaluating outcomes at 6 months demonstrated low heterogeneity (*I*^2^ = 0%), so the findings from the fixed-effect model are presented.

#### Adverse events

No adverse events were observed in any of the studies, so odds ratios could not be calculated.

The number of studies included in this systematic review was below the threshold to allow for conducting meta-regressions and funnel plot analysis.

The results of the sensitivity analyses are presented in Supplementary Table 1.

## Discussion

### Summary of evidence

The findings of the present systematic review and meta-analyses indicate that ICG-PDT produces statistically significant improvements in treatment outcomes at 3 months and 6 months post-therapy, when compared with NSPT without adjunctive treatment. For PPD, a mean additional reduction of 1.17 mm and 1.06 mm was seen at 3 and 6 months, respectively. For CAL, a mean additional gain of 0.70 mm and 1.03 mm was seen at 3 and 6 months, respectively. No adverse effects were observed in patients where ICG-PDT was administered as an adjunct.

### Level of evidence

Whilst all trials were of randomised controlled design, those included in the meta-analyses were not equal with regard to the risk of bias assessment with one deemed to be low risk, five unclear, and one high risk.

With regard to the methodology used in the trials, ‘unclear’ risk of bias assessment was made for several studies with regard to random sequence generation, allocation concealment, and blinding of outcome assessment. This was due to a lack of reporting on how these parameters were addressed in the design of the study, and due to this ambiguity, ‘unclear’ risk of bias was assigned for many studies with regard to these three parameters. Furthermore, four of the trials reported patients were lost to follow-up, and reasons for loss of patients were not clearly outlined, leading to an ‘unclear’ risk of bias assessment for these trials. A single trial was evaluated to be at ‘high’ risk of bias, and this was for the blinding of outcome assessment parameter, as the authors explicitly reported that the outcome assessors were not blinded to treatment [39]. Inclusion of this trial within the meta-analyses could have introduced bias to the results, and this was addressed through conducting sensitivity analyses. The findings of the sensitivity analyses demonstrated that when the study at high risk of bias was excluded from the meta-analyses, there was no change in the statistical significance of the overall effect size. This indicates that, despite being at high risk of bias, this study did not significantly affect the overall effect sizes observed in the meta-analyses.

### Comparison with other studies and reviews

Whilst there are no existing systematic reviews evaluating the efficacy of ICG-PDT as an adjunct to periodontal therapy, this review is supported by the existing literature which suggests that ICG-PDT is effective in eliminating periodontal pathogens, as well as evidence which suggests that ICG-PDT may convey anti-inflammatory properties [22, 23, 42]. The cytotoxic effects on causative microbial agents, combined with anti-inflammatory properties, may explain the improved treatment outcomes which are observed with adjunctive ICG-PDT. The downregulatory effects of ICG-PDT on inflammatory mediators, such as tumour necrosis factor-α, nitric oxide, and 5-lipoxygenase, have been documented, and these same inflammatory mediators are heavily implicated in the pathophysiology of periodontal disease [43–45].

The findings of this study conflict with existing reviews which have found aPDT to be of little clinical benefit in the management of periodontitis [25, 26]. However, this is explained by the fact that these existing systematic reviews synthesised the literature prior to the inception of ICG as a photosensitiser for periodontitis, and therefore, their conclusions are based off the results observed with suboptimal photosensitisers, such as toluidine blue and methylene blue. Taking the findings from the present systematic review, alongside those from previous reviews of aPDT, would indicate that ICG is more effective than the previously reviewed agents in the management of periodontitis.

In comparison with other adjunctive agents, the findings of this review indicate that adjunctive ICG-PDT produces improvements in treatment outcomes greater than those observed in systematic reviews of adjunctive systemic antimicrobials, such as amoxicillin and metronidazole, administered alone or in combination [46, 47]. Furthermore, the improvements in treatment outcomes observed in this systematic review were also greater than those observed in systematic reviews of other local adjunctive agents, such as metronidazole chlorhexidine, doxycycline, and minocycline [9, 48, 49]. Therefore, this indicates that adjunctive ICG-PDT may provide improvements in clinical outcomes equal to, or greater than, those of adjunctive antibiotics, without the same risk of developing antimicrobial resistance.

Overall, there is biological plausibility for a causative mechanism linking ICG-PDT with improved clinical outcomes, which is made up of two primary components: (i) the effects of ICG-PDT microbiota and (ii) the effects of ICG-PDT on the host immune response. The high antimicrobial efficacy of ICG-PDT with regard to periodontal pathogens such as *P. gingivalis*, which are known to be key mediators in the aetiology and pathogenesis of periodontitis [23], is critical in explaining the actions of ICG-PDT. This mechanism of action was further investigated within two of the trials included in this systematic review which also assessed microbiological outcomes [37, 38], and in both of these trials, a significant decrease in the number of Gram-negative colony-forming units was seen at sites treated with ICG-PDT. Additionally, another one of the included trials investigating cell viability found that sites treated with ICG-PDT had significantly fewer viable cells present, which further highlights the potent cytotoxic effects of the treatment modality. As periodontitis is a condition mediated by host-bacteria interactions [1], it is unlikely that all of the effects of ICG-PDT can be explained by its antimicrobial properties and more research is needed which investigates the immunomodulatory effects of ICG-PDT. However, the current evidence suggests that mediation of the immune response is a pathway through which ICG exerts its effects, and it is important that in vivo investigations in patients with periodontitis can support these findings.

### Limitations

Whilst the authors endeavoured to locate all relevant studies, it is acknowledged that there may have been studies which were not published, registered, or presented.

One of the primary limitations of this review is the quantity of evidence, both in terms of the number of trials and number of participants within trials. Across the seven trials which were suitable for meta-analysis, 237 participants were enrolled, and this sample size may be of inadequate power to allow the findings to be extrapolated to general population. Furthermore, this sample size was further reduced within the meta-analyses, as not all of the trials reported outcomes for 3 months and 6 months post-therapy; therefore, not all of the studies could be incorporated for each meta-analysis. There was significant heterogeneity for a number of the meta-analyses, and this may be attributed to the great deal of variation between the methodologies implemented in each trial. No standardised protocols have been developed for aPDT, and this is evident in the methods implemented with the trials:

- Photosensitiser concentration: three of the trials used ICG of 1 mg/ml concentrations, three used a 5mg/ml concentration, and one did not specify the concentration.
- Incubation time: three of the trials allowed the photosensitiser to stay in the pocket for 1 min prior to rinsing and irradiation, one trial specified 2 min, and one trial specified 3 min, whilst the remaining two trials did not report on the exact time.
- Light source: all trials used lasers of 810 nm wavelength; however, three of the trials administered the laser activation for 60s, two for 30s, one for 40s, and one for 5s.
- Rinsing protocol: all trials washed out excess photosensitiser from the pocket prior to irradiation.
- Irradiation method: two of the trials specified direct irradiation of the pockets, two of the trials specified combination of direct and transcutaneous irradiation, and two did not report on the exact irradiation protocol.
- Number of sessions: six of the trials evaluated a single session of ICG-PDT, whilst one evaluated one session with three follow-up sessions.
- Timing of sessions: five of the trials initiated ICG-PDT immediately following the completion of NSPT, whilst two left one week between NSPT and aPDT. Furthermore, the trial which utilised additional follow-up sessions carried these out at 7, 17, and 27 days after the first session.

It is critical that these factors are standardised as they are known to have quantifiable effects on the outcomes of aPDT: ICG is known to display different effects at different concentrations and has varying absorption rates, meaning it is key to understand how it governs clinical outcomes at a range of different concentrations [50, 51]; different incubation times within the pocket may affects outcomes as it is currently unknown what the optimal time for incubation is and whether ICG may display negative effects at times which are shorter or greater than this; factors relating to the light source (e.g. wavelength, time, irradiation method) are also key as different irradiation times are known to have differential effects on oral microbiota, and in addition, most of the cytotoxic activity of aPDT occurs in the superficial layers of the biofilm due to inadequate light penetration, meaning that longer irradiation times and the use of direct irradiation could affect outcomes [52–54].

Additionally, details of the subgingival debridement methodology were not described across the trials, which may have introduced further heterogeneity.

Furthermore, outcomes were not evaluated over extensive time periods. Longer follow-up periods are needed before judgements on the long-term effectiveness of ICG-PDT can be made.

In order to allow for more accurate pooling of data, it would be advised that future researchers:

- Enrol a greater number of participants into randomised controlled trials
- Implement methods to minimise risk of bias, such as allocation concealment and blinding (where feasible)
- Develop and use a standardised protocol for the administration of ICG-PDT
- Develop and use a standardised protocol for the administration of NSPT
- Evaluate outcomes over a longer time period

## Conclusion

Within the limitations of this study, it can be concluded that:

- Adjunctive ICG-PDT may produce significant improvements in clinical outcomes of NSPT.
- Adjunctive ICG-PDT does not increase the risk of adverse events.
- More high-quality, randomised controlled trials are necessitated before recommendations for use can be made.

## Supplementary information

ESM 1 (DOCX 13 kb)

ESM 2 (DOC 64 kb)
