# Supplementary material for: Indocyanine green–mediated antimicrobial photodynamic therapy as an adjunct to periodontal therapy: a systematic review and meta-analysis
Source: Clin Oral Investig. 2021 Mar 12;25(10):5699–710. doi: 10.1007/s00784-021-03871-2 (PMC8443506; doi:10.1007/s00784-021-03871-2)
Supplement: Supplementary file 1 — (DOCX 13 kb) [file 784_2021_3871_MOESM1_ESM.docx]

**Table S1.** Sensitivity analysis for comparison of ICG-PDT with NSPT and no laser therapy

| **Outcome Measure** | **Study Excluded** | **New Observed Effect** |
| --- | --- | --- |
| PPD reduction (3 months) | Gandhi et al. 2019 | 1.08 mm (95% CI: 0.42 – 1.75 mm) |
|  | Joshi et al. 2019 | 1.37 mm (95% CI: 0.93 – 1.81 mm) |
|  | Monzavi et al. 2016 | 1.01 mm (95% CI: 0.46 – 1.56 mm) |
|  | Sethi et al. 2019 | 1.17 mm (95% CI: 0.50 – 1.84 mm) |
|  | Shingnapurkar et al. 2016 | 1.15 mm (95% CI: 0.45 – 1.84 mm) |
|  | Srikanth et al. 2015 | 1.24 mm (95% CI: 0.47 – 2.01 mm) |
| PPD reduction (6 months) | Gandhi et al. 2019 | 1.09 mm (95% CI: 0.27 – 1.90 mm) |
|  | Raut et al. 2018 | 0.73 mm (95% CI: 0.51 – 0.94 mm) |
|  | Srikanth et al. 2015 | 1.30 mm (95% CI: 0.80 – 1.80 mm) |
| CAL gain (3 months) | Gandhi et al. 2019 | 0.53 mm (95% CI: 0.05 – 1.01 mm) |
|  | Joshi et al. 2019 | 0.80 mm (95% CI: 0.17 – 1.43 mm) |
|  | Monzavi et al. 2016 | 0.88 mm (95% CI: 0.33 – 1.43 mm) |
|  | Sethi et al. 2019 | 0.74 mm (95% CI: 0.17 – 1.31 mm) |
|  | Shingnapurkar et al. 2016 | 0.58 mm (95% CI: 0.05 – 1.11 mm) |
|  | Srikanth et al. 2015 | 0.65 mm (95% CI: 0.04 – 1.25 mm) |
| CAL gain (6 months) | Gandhi et al. 2019 | 1.05 mm (95% CI: 0.83 – 1.26 mm) |
|  | Raut et al. 2018 | 1.05 mm (95% CI: 0.83 – 1.28 mm) |
|  | Srikanth et al. 2015 | 0.94 mm (95% CI: 0.55 – 1.33 mm) |
